# Identification and Biosynthesis of Novel Male Specific Esters in the Wings of the Tropical Butterfly, ***Bicyclus martius sanaos***

**DOI:** 10.1007/s10886-014-0452-y

**Published:** 2014-06-04

**Authors:** Hong-Lei Wang, Oskar Brattström, Paul M. Brakefield, Wittko Francke, Christer Löfstedt

**Affiliations:** 1 Department of Biology, Lund University, 223 62 Lund, Sweden; 2 Department of Zoology, Cambridge University, Cambridge, UK; 3 Institute of Organic Chemistry, University of Hamburg, Hamburg, Germany

**Keywords:** Pheromone, Biosynthesis, Δ11-desaturase, Amino acid, Butterfly, *Bicyclus martius sanaos*, Lepidoptera

## Abstract

Representatives of the highly speciose tropical butterfly genus *Bicyclus* (Lepidoptera: Nymphalidae) are characterized by morphological differences in the male androconia, a set of scales and hair pencils located on the surface of the wings. These androconia are assumed to be associated with the release of courtship pheromones. In the present study, we report the identification and biosynthetic pathways of several novel esters from the wings of male *B. martius sanaos*. We found that the volatile compounds in this male butterfly were similar to female-produced moth sex pheromones. Components associated with the male wing androconial areas were identified as ethyl, isobutyl and 2-phenylethyl hexadecanoates and (11*Z*)-11-hexadecenoates, among which the latter are novel natural products. By topical application of deuterium-labelled fatty acid and amino acid precursors, we found these pheromone candidates to be produced in patches located on the forewings of the males. Deuterium labels from hexadecanoic acid were incorporated into (11*Z*)-11-hexadecenoic acid, providing experimental evidence of a Δ11-desaturase being active in butterflies. This unusual desaturase was found previously to be involved in the biosynthesis of female-produced sex pheromones of moths. In the male butterflies, both hexadecanoic acid and (11*Z*)-11-hexadecenoic acid were then enzymatically esterified to form the ethyl, isobutyl and 2-phenylethyl esters, incorporating ethanol, isobutanol, and 2-phenylethanol, derived from the corresponding amino acids L-alanine, L-valine, and L-phenylalanine.

**Electronic supplementary material:**

The online version of this article (doi:10.1007/s10886-014-0452-y) contains supplementary material, which is available to authorized users.

## Introduction

In the insect order Lepidoptera, moths and butterflies differ in their use of pheromones for mate finding and mate recognition. Mate finding in moths is mediated by female-emitted sex pheromones that elicit a fine-tuned response in conspecific males (Wyatt 2003). Pheromone biosynthesis in most moths involves a series of more or less well-described enzymatic reactions, including *de novo* synthesis of fatty acyl precursors, desaturation, and chain elongation or shortening to produce various carbon backbones, followed by transesterification to form corresponding fatty acid esters. Reduction leads to alcohols, which in turn may yield acetates or aldehydes after re-esterification or oxidation, respectively (Francke and Schulz 1999; Tillman et al. 1999). So far there is no evidence for sex pheromones to be involved in long-range communication in butterflies: they appear to have lost this information channel used by female moths. However, male-produced pheromones are commonly involved in close-range courtship displays (Vane-Wright and Boppré 1993). Detailed studies on the semiochemicals of butterflies include Nymphalids e.g. some Milkweed butterflies from the subfamily Danainae (Nishida et al. 1996; Schulz et al. 1993, 2004; Stritzke et al. 2003) and some *Heliconius* spp. from the subfamily Heliconiinae (Schulz et al. 2007, 2008), and Pierid butterflies such as *Colias eurytheme* and *C. philodice* from the subfamily Coliadinae (Grula et al. 1980; Rutowski 1980), and *Pieris napi* (Andersson et al. 2007; Bergström and Lundgren 1973), *P. rapae* and *P. brassicae* (Andersson et al. 2003; Yildizhan et al. 2009) from the subfamily Pierinae. In contrast to moths that generally use *de novo* synthesised compounds to produce volatile signals, butterflies often utilize chemical substances acquired from plants in scent production (Boppré 1984; Schulz et al. 2004).

The tropical butterfly genus *Bicyclus* (Lepidoptera: Nymphalidae) is highly speciose, with over 90 species in Africa, and species determination is sometimes challenging. The most important classical taxonomic characters for the genus are differences in the male androconia (Condamin 1973), a set of differentiated scales and hair pencils located on the surface of the wings and assumed to be associated with the release of pheromones. Male-produced courtship pheromones have been identified from the squinting bush brown, *B. anynana* (Nieberding et al. 2008). In this species, volatile signals are as important as visual cues in female choice (Costanzo and Monteiro 2007), and are most likely involved in mate quality assessment (Nieberding et al. 2012; Van Bergen et al. 2013). Over the past 5 years we carried out a large-scale analysis of chemicals found in more than 30 species of *Bicyclus* (Bacquet P.M.B., Brattström O., Wang H.L., Allen C.E., Löfstedt C., Brakefield P.M. and Nieberding C.M. unpublished data). Our results strongly suggest that the use of male pheromones is generalized within the genus, and that the pheromone signals are species-specific. Among all the candidate pheromone components in the genus *Bicyclus*, nearly two thirds of the compounds are chemically classified as saturated and unsaturated fatty alcohols, aldehydes, esters, and hydrocarbons, which are structurally similar to female-produced moth pheromones, suggesting a close biosynthetic relationship with common moth pheromone components.

To test the hypothesis that conserved pheromone biosynthetic pathways and related enzymes/genes are used in both moths and butterflies, we carried out biosynthesis studies of volatile compounds in representative *Bicyclus* species. In the present study, we report the identification of several novel ethyl, isobutyl, and 2-phenylethyl esters in *B. martius sanaos.* We also investigated the origin of the alcohols and the biosynthesis of the unsaturated fatty acyl moiety involving a Δ11-desaturase, an enzyme family that has previously been found typically in relation with moth sex pheromone biosynthesis.

## Methods and Materials

### Insects

A laboratory colony of *B. martius sanaos*, originally started in 2009 from around 50 adult females collected in Ologbo Forest (N 6.02, E 5.55, 20 m.a.s.l.) in southern Nigeria, was maintained at 27 ± 1 °C and 70 % relative humidity under a 12:12 hr L:D photoperiod. The larvae of different instars were separately fed on the leaves of potted wheat, and pupated directly on the wheat stems at the end of their larval stage. Newly emerged adults were collected daily and held in single-sex cohorts feeding on banana. Adult males (7-d to 10-d-old) were used for chemical analysis and labelling experiments. In the labelling experiment, the adults were fed on 10 % glucose solution in order to avoid the possible involvement of food-derived substances.

### Chemicals

Ethyl and butyl esters of hexadecanoic acid, (11*E*)-11-hexadecenoic acid and (11*Z*)-11-hexadecenoic acid were prepared by mixing the acids separately with ethanol or each of the butanol isomers (*n*-, *sec*-, *iso*-, and tert-) in HCl (0.5 M) in a ratio of 1:1.2 (acid : alcohol). The reaction was run at 80 °C for 1 hr and stopped by adding saturated aqueous sodium carbonate solution. The target ester was extracted with hexane. To prepare the 2-phenylethyl esters, the acids were separately mixed with 2-phenylethyl alcohol in a ratio of 1:1.2, and the reaction was carried out at 120 °C for 2 hr after adding one drop of concentrated sulfuric acid. The two unsaturated acids were prepared by oxidation of corresponding alcohols, (11*E*)-11-hexadecen-1-ol and (11*Z*)-11-hexadecen-1-ol from our laboratory stock with pyridinium dichromate (PDC) in dimethylformamide (DMF) at 25 °C for 10 hr. The synthesis of (11*Z*)-13,13,14,14,15,15,16,16,16-^2^H_9_-11-hexadecenoic acid- (*d*_9_-*Z*11-16:Acid) has been described in Löfstedt et al. (1994). Deuterium substituted 16,16,16-^2^H_3_-hexadecanoic acid (*d*_3_-16:Acid), 2,3,3,3-^2^H_4_-L-alanine (*d*_4_-Ala), 2,3,4,4,4,4′,4′,4′-^2^H_8_-L-valine (*d*_8_-Val), and L-phenyl-^2^H_5_-alanine-2,3,3-^2^H_3_ (*d*_8_-Phe) were purchased from Cambridge Isotope Lab (Larodan Fine Chemicals, Limhamn and Malmö, Sweden).

### Preparation of Extracts

For the initial investigation of the compounds found in individual androconia of *B. martius sanaos*, butterflies were collected in the field at the location where collections were made for setting up the laboratory colony. Traps baited with fermented bananas or hand netting were used. Three males and two females were sampled individually. The thorax was pinched prior to sampling, and then samples of different part of the wings, i.e., the patch-like tissue and several brush-like androconia (Fig. S1), were cut out using micro-scissors cleaned in 99 % alcohol between each cut to avoid contamination across samples. All sampling were done within 15 min to minimize possible post-mortem changes to the chemical composition. The wing tissue samples then were extracted individually in a 1.5 ml glass vial with 100 μl of redistilled heptane containing 1 ng/μl of (8*Z*)-8-tridecen-1-yl acetate as internal standard. After the androconia had been removed, the remaining wing tissue of the hind and forewing were extracted separately with 300 μl of heptane containing the same internal standard in a concentration of 0.33 ng/μl.

For the biosynthesis study, butterflies from our laboratory population were used. The analyses of wing esters and biosynthetic precursors were repeated in five individuals. Fore and hindwings of unmated males were collected 4 hr before the scotophase when courtship behavior was observed, and extracted in 200 μl hexane for 30 min. Thorax and abdomen tissue from the same individuals were dissected and separately extracted in hexane. After the hexane extraction, the fatty acids in the remaining tissues were further extracted with 200 μl chloroform:methanol (2:1 *v*:*v*) at room temperature for 24 hr. The chloroform-methanol extracts were subjected to base methanolysis to convert fatty acyl moieties to the corresponding methyl esters as described by Bjostad and Roelofs (1984).

Samples were analysed by coupled gas chromatography/mass spectrometry (GC/MS), and compounds were identified based on comparison of their retention times and mass spectra with those of synthetic references on both polar and non-polar columns. Double bond positions in unsaturated esters and fatty acid precursors were localized by analysis of the dimethyldisulphide (DMDS) derivatization adducts, prepared according to Dunkelblum et al. (1985).

### Labelling Experiment

Deuterium substituted amino acids (*d*_4_-Ala, *d*_8_-Val, *d*_8_-Phe), and fatty acids (*d*_3_-16:Acid, *d*_9_-*Z*11-16:Acid) were dissolved in dimethylsulfoxide (DMSO) at a concentration of 20 μg/μl and topically applied to trace the biosynthetic pathways leading to the esters. Both intact individuals and males in which the forewings or hindwings had been removed were used for labelling, in order to clarify the potential biosynthetic site. The solution of a labelled precursor (1 μl) was topically applied 4 hr before the scotophase to the tentative biosynthetic site on the wings, i.e., the brush-like androconium on the hindwing, or the patch-like tissue on forewing. Thus, *d*_9_-*Z*11-16:Acid was topically applied on: 1) the forewings of individuals in which the hindwings had been removed beforehand, 2) the hindwings of individuals in which the forewings had been removed beforehand, and 3) the left forewing and right hindwing of the same individual, in which the left hindwing and right forewing had been removed beforehand. After 24-hr incubation, the wings were extracted in 200 μl of hexane for 30 min. The extracts were analysed by GC/MS as described below. Individuals treated with DMSO solution alone were extracted in parallel as a control.

### Coupled Gas Chromatography/Mass Spectrometry

The wing extracts were analyzed with an Agilent 5975 mass spectrometer coupled to an Agilent 6890 GC and an HP-5MS or INNOWax capillary column (30 m × 0.25 mm i.d., Agilent Technologies, USA). The oven temperature was programmed from 80 °C, held for 1 or 3 min, then programmed to 210 °C at 10 °C/min and held for 12 min, and finally to 250 °C at 10 °C/min and held for 5 min. The DMDS derivatives were analyzed on the HP-5MS column under a programmed conditions starting from 80 °C, held for 2 min, then to 140 °C at 20 °C/min, and finally to 270 °C at 4 °C/min and held for 30 min. For the labelling experiments, the native and deuterated male wing-esters and fatty acid precursor were monitored by the selected ions shown in Table 1.

**Table 1:** Fragment ions for monitoring unlabelled and deuterated esters and fatty acid precursors (16:EE ethyl hexadecanoate; Z11-16:EE ethyl (11*Z*)-11-hexadecenoate; 16:iBE isobutyl hexadecanoate; Z11-16:iBE isobutyl (11Z)-11-hexadecenoate; 16:PEE 2-phenylethyl hexadecanoate; Z11-16:PEE 2-phenylethyl (11*Z*)-11-hexadecenoate; Z11-16:ME methyl (11*Z*)-11-hexadecenoate)

Precursor	Selected ions*							
	16:EE	Z11-16:EE	16:iBE	Z11-16:iBE	16:PEE	Z11-16:PEE	16:Me	Z11-16:ME
Unlabelled	88, **101** ^*^, 239, **284**	88, **237**, 282	**239**, **312**	**237**, **310**	104, **105**, 360	237, 104, **105**, 358	74, **270**	74, **236**, 268
*d*_3_-16:Acid labeled	88, 242, **287**	88, **240**, 285	**242**, 315	**240**, 313	104, 105, 363	104, 105, 361	74, **273**	74, **239**, 271
*d*_9_-*Z*11-16:Acid labeled		88, **246**, 291		**246**, 319		104, 246, 367		
*d*_4_-Ala labeled	91, 92, **104**, 105, 239, 287, 288	91, 92, **104**, 105, 237, 285, 286						
*d*_8_-Val labeled			239, **319**	237, **317**				
*d*_8_-Phe labeled					111, **112**, 367	111, **112**, 365		

^*^Ions in bold are shown in Fig. 4

## Results

### Structure Elucidation of Volatile Compounds

**Fig. 1:**
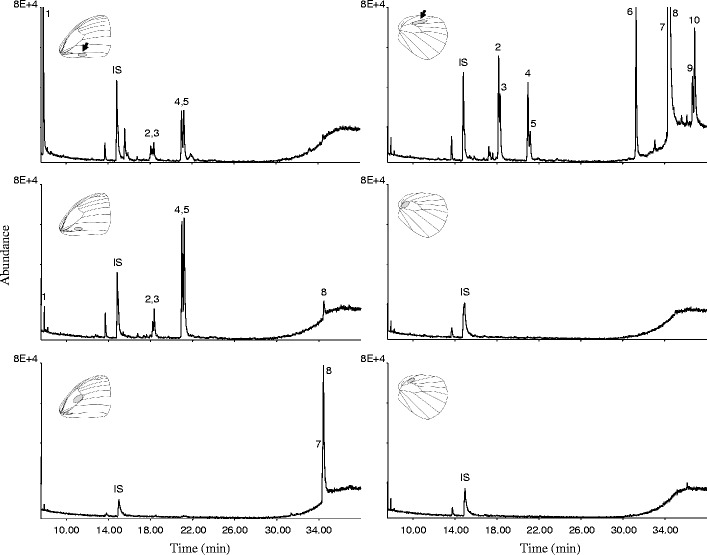
Representative total ion chromatogram (TIC) recorded by GC/MS analysis of male wings of *Bicyclus martius sanaos.* The insert pictures show the sampling positions as grey circles on the forewings (*left*) and hindwings (*right*). *Black arrows* indicate the positions of the patch-like tissue on forewing and brush on hindwing, on which the labeled precursors were applied. (HP-5MS column; 1 ethyl benzoate; 2 ethyl (11*Z*)-11-hexadecenoate; 3 ethyl hexadecanoate; 4 isobutyl (11*Z*)-11-hexadecenoate; 5 isobutyl hexadecanoate; 6 2-phenylethyl tetradecanoate; 7 2-phenylethyl (11*Z*)-11-hexadecenoate; 8 2-phenylethyl hexadecanoate; 9 2-phenylethyl octadecenoate; 10 2-phenylethyl octadecanoate; IS internal standard, (8*Z*)-8-tridecen-1-yl acetate)

GC/MS analysis (HP-5 column) of solvent extracts from various body parts of adult males and females revealed a number of male-produced, wing-specific compounds (Fig. 1). The occurrence of these compounds differed between various parts of the wings, and was most abundant in the patch-like tissue on the forewings and the brush on the hindwings (Fig. 1, peak 1–10, Fig. S1). The absolute amounts varied significantly between different components, ranging from ca. 400 to 8,000 ng per male, whereas the relative amounts of the key components were consistent between individuals.

Compound 1 exhibited a mass spectrum identical to ethyl benzoate (NIST 2002), which was verified by comparing its mass spectrum and GC retention time with corresponding data of a reference compound on both polar and non-polar columns.

**Fig. 2:**
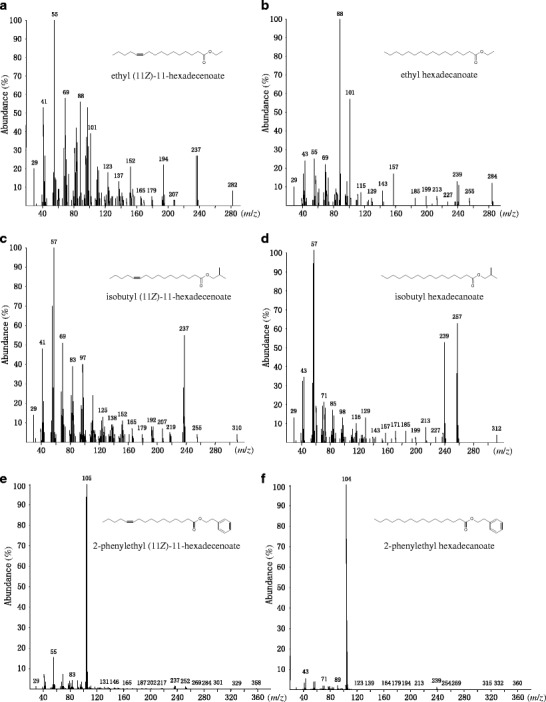
Mass spectra of the major esters from wings of male *Bicyclus martius sanaos*. **a** ethyl (11*Z*)-11-hexadecenoate. **b** ethyl hexadecanoate. **c** isobutyl (11*Z*)-11-hexadecenoate. **d** isobutyl hexadecanoate. **e** 2-phenylethyl (11*Z*)-11-hexadecenoate. **f** 2-phenylethyl hexadecanoate

The mass spectrum of compound 3 showed a likely molecular ion at *m/z* 284, an abundant McLafferty fragment at *m*/*z* 88, and a peak at *m/z* 101 (classical β-cleavage) as diagnostic signals of the ethyl ester of hexadecanoic acid (Fig. 2b), which was confirmed by comparison of the mass spectrum and retention times of the natural product with corresponding data for synthetic ethyl hexadecanoate on two columns. The mass spectrum of compound 2 (Fig. 2a) showed a likely molecular ion at *m*/*z* 282, a pair of signals at *m/z* 237 and *m/z* 236 produced by the acylium ion and the corresponding ketene (Francke et al. 2000), as well as relatively less abundant but still apparent fragments at *m*/*z* 88 and 101 as diagnostic signals of the ethyl ester of a hexadecenoic acid. The spectrum of the DMDS adduct (Dunkelblum et al. 1985) revealed a double bond position between carbon atoms 11 and 12 of the acyl skeleton by displaying a diagnostic ion pair at *m/z* 117/259, and a molecular ion at *m*/*z* 376 (Fig. S2a). Comparison of gas chromatographic retention times of compound 2 with *Z*- and *E*-isomers of ethyl 11-hexadecenoate (co-injection) proved compound 2 had the 11*Z*-configuration.

The mass spectrum of compound 5 (Fig. 2d) exhibited a likely molecular ion at *m*/*z* 312, and a McLafferty ion at *m*/*z* 116, as well as two intense signals at *m*/*z* 239 and 257, suggesting a butyl ester of hexadecanoic acid. The fragments at *m*/*z* 239 and 257 reflect the formation of a hexadecanoylium ion by loss of a butyloxy group (M-OC_4_H_9_) and that of the protonated acid, respectively (Francke et al. 2000). A base peak at *m*/*z* 57 instead of 56 in the mass spectrum implied a branched, rather than a straight butyl group forming the ester moiety. Indeed, compound 5 proved to be isobutyl hexadecanoate as shown by comparison of retention times and mass spectra of the four possible butyl-hexadecanoates with corresponding data for the natural product on both columns. The mass spectrum of compound 4 (Fig. 2c) indicated a likely molecular ion at *m*/*z* 310, diagnostic fragments at *m/z* 255, *m*/*z* 237, and *m/z* 236 as well as a base peak at *m*/*z* 57, suggesting the butyl ester of a hexadecenoic acid. The spectrum of the DMDS adduct (Fig. S2b) revealed a double bond at position 11 by displaying fragments at *m*/*z* 117/287, and a molecular ion at *m*/*z* 404 (Dunkelblum et al. 1985). Compound 4 was confirmed to be isobutyl (11*Z*)-11-hexadecenoate by comparing its retention times and mass spectrum with corresponding data for the synthetic *Z*- and *E*-isomers of isobutyl 11-hexadecenoate on two columns.

The fragmentation pattern of compounds 6–10 were similar: An abundant styryl related fragment (ArCH=CH_2_)^+^ at *m*/*z* 104 was apparent, along with another dominating signal (ArCH_2_CH_2_)^+^ at *m*/*z* 105, whereas other peaks in the mass spectra were of only very low abundance. This pattern suggested the compounds to be 2-phenylethyl esters of carboxylic acids (Emery 1960). The fragment at *m*/*z* 239 in the spectrum of compound 8 (Fig. 2f), in analogy to compounds 3 and 5, suggested a hexadecanoylium ion formed by cleavage of the carbon-oxygen bond of a corresponding ester. A tentative molecular ion at *m*/*z* 360, present in the spectrum, further supported the structure of 2-phenylethyl hexadecanoate, which was confirmed by comparison with a synthetic reference compound. The mass spectrum of compound 7 closely resembled that of compound 8, showing most abundant signals at *m*/*z* 105 and *m*/*z* 104 (Fig. 2e). In addition, the diagnostic fragment at *m*/*z* 237 and the molecular ion at *m*/*z* 358 were visible, suggesting 2-phenylethyl hexadecenoate as the target structure. The spectrum of the DMDS adduct of compound 7 (Fig. S2c), displaying fragments at *m*/*z* 117 and *m*/*z* 335 and a molecular ion at *m*/*z* 452, revealed a Δ11-double bond position in the hexadecenoyl skeleton, similar to that in compounds 2 and 4. Comparison of gas chromatographic retention times of the natural product with data for the *Z*- and *E-*isomers of 2-phenylethyl 11-hexadecenoate proved the double bond to have *Z-*configuration.

The mass spectra of compounds 6, 9, and 10 again exhibited similar fragmentation patterns (Fig. S3) and showed close relations to those of compounds 7 and 8. In the spectra of compounds 6 and 10 (Fig. S3a, b), two homologous acyl fragments at *m*/*z* 211 (tetradecanoyl) resp. 267 (octadecanoyl) were found, implying the compounds to be 2-phenylethyl tetradecanoate and 2-phenylethyl octadecanoate, respectively, although the expected molecular ions at *m*/*z* 332 and *m*/*z* 388 could not be detected. The structures were verified by using authentic reference compounds. In the mass spectrum of compound 9 (Fig. S3c), the signal at *m*/*z* 265, corresponding to a 2C-bishomologue of *m/z* 237 in compound 7 suggested 2-phenylethyl octadecenoate as the target structure. However, due to low signal intensities in the mass spectrum of the natural product, the expected molecular ion at *m*/*z* 386 could not be seen, and the position and configuration of the double bond could not be determined.

### Δ11-Desaturation and *In Vivo* Label Incorporation

**Fig. 3:**
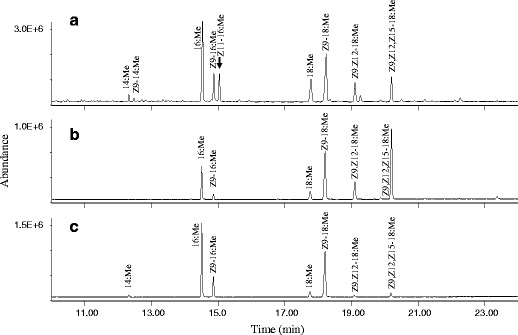
GC/MS Analyses of fatty acid methyl esters derived from **a** wings, **b** thorax and **c** abdomen of male *Bicyclus martius sanaos*. (INNOWax column; 14:Me methyl tetradecanoate; *Z*9-14:Me methyl (9*Z*)-9-tetradecenoate; 16:Me methyl hexadecanoate; *Z*9-16:Me methyl (9*Z*)-9-hexadecenoate; *Z*11-16:Me methyl (11*Z*)-11-hexadecenoate; *Z*9-18:Me methyl (9*Z*)-9-octadecenoate; *Z*9,*Z*12-18:Me methyl (9*Z*,12*Z*)-9,12-octadecadienoate; *Z*9,*Z*12,*Z*15-18:Me methyl (9*Z*,12*Z*,15*Z*)-9,12,15-octadecatrienoate)

The fatty acid compositions of the chloroform/methanol extracts of wings, abdomen, and thorax tissue of male adults were analyzed as methyl esters after methanolysis (Fig. 3a–c). An unusual hexadecenoic acid was found exclusively in the male wing extracts, along with a number of common fatty acids including palmitic, palmitoleic, stearic, oleic, linoleic, and linolenic acids that were constantly present in all the analyzed tissues (Fig. 3a). The DMDS adduct of the unusual methyl hexadecenoate exhibited a diagnostic pair of fragments at *m/z* 117/245 and a molecular ion at *m*/*z* 362, revealing a Δ11-double bond position as reported by Dunkelblum et al. (1985). The double bond was confirmed to have *Z*-configuration by comparing the retention times and mass spectrum of the target compound with data of synthetic methyl *Z*- and *E*-11-hexadecenoate. A further comparison of extracts from various locations on the male wings showed this acid to be widely present in both forewings and hindwings (data not shown). In addition, small amounts of methyl (7*Z*)-7-hexadecenoate, with the position and geometry of the double bond confirmed by its DMDS-adduct, were also found in the male wing extracts.

**Fig. 4:**
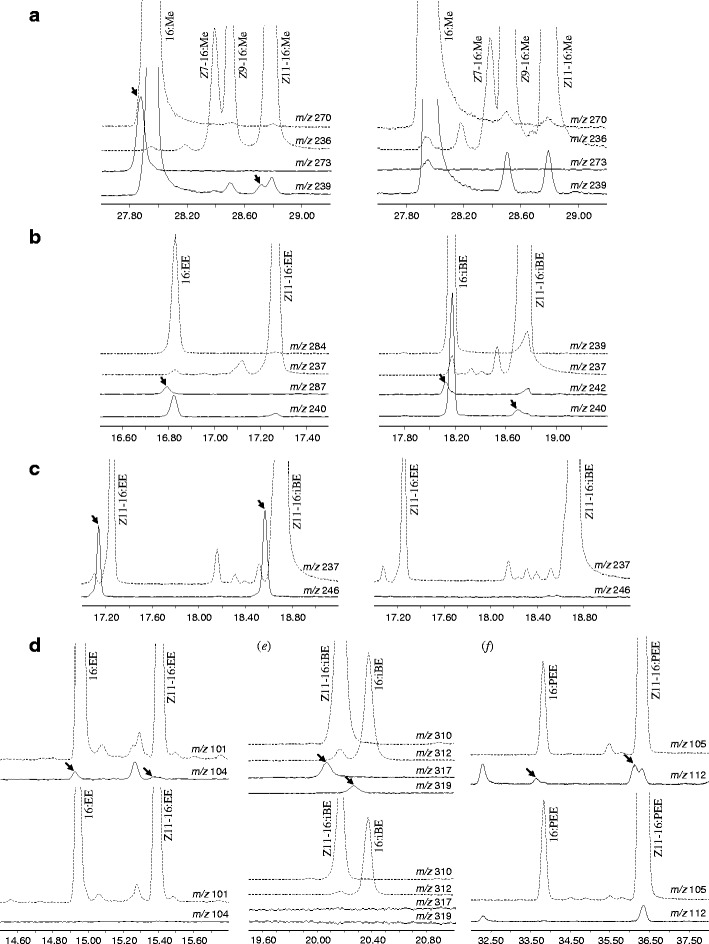
GC/MS analyses of esters from wings of male *Bicyclus martius sanaos* showing incorporation of deuterium labels from fatty acid and amino acid precursors (Selected ion monitoring (SIM) with INNOWax column except for (**e**) with HP5-MS; unlabelled components shown by *dashed lines*, corresponding deuterium-labelled compounds by *solid lines* and indicated by *arrows*): **a** incorporation from saturated hexadecanoic acid (*d*_3_-16:Acid) into the monounsaturated (11*Z*)-11-hexadecenoic acid (Z11-16:Acid) (*left*) compared with the treatment of DMSO solvent control (*right*) analysed as methyl esters; **b** incorporation from *d*_3_-16:Acid into ethyl hexadecanoate (16:EE), isobutyl hexadecanoate (16:iBE) and isobutyl (11*Z*)–hexadecenoate (Z11-16:iBE); **c** incorporation from *d*_9_-Z11-16:Acid into ethyl (11*Z*)-11-hexadecenoate (Z11-16:EE) and Z11-16:iBE in forewings (*left*) but not in hindwings (*right*); **d** incorporation of *d*_4_-Ala into 16:EE and Z11-16:EE in forewings (*top*) but not in hindwings (*bottom*); **e** incorporation of *d*_8_-Val into 16:iBE and Z11-16:iBE in forewings (*top*) but not in hindwings (*bottom*); **f** incorporation of *d*_8_-Phe into 16:PEE and Z11-16:PEE in forewings (*top*) but not in hindwings (*bottom*)

When deuterium substituted *d*_3_-16:Acid was topically applied on the wings of intact male adults, incorporation of all three deuterium atoms into (11*Z*)-11-hexadecenoic acid was observed, but no label was incorporated into (9*Z*)-9-hexadecenoic acid (palmitoleic acid) (Fig. 4a). The three deuterium atoms from *d*_3_-16:Acid also were incorporated into both saturated and unsaturated ethyl and isobutyl esters (Fig. 4b).

Topical application of *d*_9_-*Z*11-16:Acid on various parts of the wings showed that all nine deuterium atoms were incorporated into ethyl and isobutyl (11*Z*)-11-hexadecenoate (Fig. 4c). Furthermore, deuterium atoms from *d*_9_-*Z*11-16:Acid were incorporated only into the forewing-extracted ethyl and isobutyl (11*Z*)-11-hexadecenoates, but not into the same esters extracted from the hindwings (Fig. 4c). In addition, very small amounts of remaining *d*_9_-*Z*11-16:Acid were found in the extract of labelled forewings after 24-h incubation, whereas high amounts of unmetabolised *d*_9_-*Z*11-16:Acid were found in the extract of hindwings treated with the labelled acid.

Because the 2-phenylethyl esters produced extremely abundant styryl related signals at *m*/*z* 104 and 105, but weak ones at all other mass units, it was impossible to monitor the deuterium-incorporation from the two aliphatic acids, *d*_3_-16:Acid and *d*_9_-*Z*11-16:Acid. On the other hand, incorporation of 7 deuterium atoms into the 2-phenylethyl group of the corresponding esters originating from *d*_8_-Phe, applied to the wings was clearly observed. Similarly, 3 and 7 deuterium atoms were incorporated into the corresponding ethyl and isobutyl esters from *d*_4_-Ala and *d*_8_-Val, respectively. The incorporation of deuterium atoms from these amino acids into the esters was observed only in the forewing extracts, but not in the hindwing extracts (Fig. 4d, e, f).

## Discussion

Males of *Bicyclus martius sanaos* produce ethyl, isobutyl, and 2-phenylethyl esters of (11*Z*)-11-hexadecenoic and hexadecanoic acid, which were not found in conspecific females. Some of these unusual compounds represent new natural products. The same esters or their structural analogs have been found also in other closely related *Bicyclus* species (Bacquet P.M.B., Brattström O., Wang H.L., Allen C.E., Löfstedt C., Brakefield P.M. and Nieberding C.M. unpublished data), and some of these *Bicyclus* esters, i.e., ethyl hexadecanoate and isobutyl hexadecanoate, as well as an ethyl hexadecenoate, in which the double bond position and configuration were not determined, have been identified previously in *Heliconius* butterflies as components of a highly complex mixture of esters, acids, and hydrocarbons. These compounds possibly act as a matrix or solvent for monoterpenes, especialy (*E*)-β-ocimene that males transfer to the females as an antiaphrodisiac during copulation (Yildizhan et al. 2009). The biosynthetic pathways yielding the esters demonstrated here for *B. martius sanaos* are probably the same in the other butterfly species that have similar compounds*.*

The combination of Δ11-desaturation with various conventional chain shortening or chain-elongation steps explains the production of most of the identified pheromones in noctuid, pyralid, and tortricid moths (Roelofs and Bjostad 1984). The unusual Δ11-desaturase was first found in the biosynthesis of the female-produced sex pheromone of the redbanded leafroller moth, *Argyrotaenia velutinana* (Bjostad and Roelofs 1981). Subsequently, a number of unique Δ11-desaturases involved in sex pheromone biosynthesis were discovered in various moth species (Roelofs and Rooney 2003). The Δ11-desaturase gene was believed to have evolved from the Δ9-desaturase gene, and was first cloned and functionally characterized from the sex pheromone gland of females of the cabbage looper, *Trichoplusia ni* (Knipple et al. 1998).

Our *in vivo* labelling experiment with the butterfly *B. martius sanaos* showed direct incorporation of the deuterium atoms from *d*_3_-16:Acid into *d*_3_-*Z*11-16:Acid, thus confirming the existence of an active Δ11-desaturase also in butterflies. In a parallel study, Liénard et al. (2014) showed that one of the pheromone components that play a major role in mate choice in *B. anynana*, (9*Z*)-9-tetradecen-1-ol, is produced through the activity of a fatty-acyl ∆11-desaturase on palmitic acid, followed by chain shortening and the action of a specialized alcohol-forming fatty-acyl reductase similar to what has previously been reported in moths. This provides evidence of conservation and sharing of ancestral genetic modules for the production of fatty acid-derived pheromones over a long evolutionary timeframe, thereby reconciling mate communication in moths and butterflies. This is after all not surprising as butterflies form a group of Lepidoptera nested within the more advanced moths.

Different labeled precursors were topically applied on the patch-like androconia on forewings and the brush-like androconia on hindwings. Deuterium-incorporation into the esters was observed exclusively in the forewing extracts, thus indicating that the patch tissue is the likely biosynthetic site, presumably containing active biosynthetic enzymes such as desaturase, esterase, and enzymes associated with amino acid metabolism. However, the key acyl intermediate, *Z*11-16:Acid, was found on both forewings and hindwings, implying either a wide tissue expression of the desaturase or an effective post-production transportation, possibly via hemolymph inside the veins. Further studies on gland histochemistry and molecular biology may provide more detailed information.

The transformation of branched-chain and aromatic amino acids into corresponding alcohols has been investigated previously in yeast in which the so-called Ehrlich pathway accounts for the process (Dickinson 2000; Hazelwood et al. 2008). According to this pathway, branched-chain amino acids such as valine, leucine, and isoleucine or aromatic amino acids such as phenylalanine and tyrosine, are converted into α-keto acids during a transamination reaction under loss of the α-hydrogen. Subsequently, the α-keto acids are decarboxylated to aldehydes, which are either oxidized to the corresponding acids or reduced to alcohols. Consequently, *d*_8_-Val and *d*_8_-Phe will furnish 2,3,3,3,3′,3′,3′-^2^H_7_-isobutanol and ^2^H_5_-2-phenyl-2,2-^2^H_2_-ethanol, respectively. This is what was found, indicating that the Ehrlich pathway is probably also present in the biosynthesis of the *Bicyclus* esters. Similarly, 2,2,2-^2^H_3_-ethanol is produced from *d*_4_-Ala, indicating that the same pathway is involved.

Insects may synthesize esters from exogenously acquired alcohols such as for the ethyl oleate found in honeybees (Castillo et al. 2012). In the present study, butterflies feeding on glucose solution showed the same ester composition as those feeding on fresh banana, indicating that adult males do not necessarily require the exogenous alcohols as ester components, but instead they can use *de novo* generated ethanol, isobutanol, and 2-phenylethanol. The selective use of alanine, valine, and phenylalanine by *B. martius sanaos* to produce the alcohols may relate to the substrate specificity of the aminotransferases and the acyltransferases involved. We suggest that this is the key factor determining the type of esters. On the other hand, whether these compounds are definitely biosynthesized *de novo* by the insects or by associated microorganisms awaits further investigations.

The involvement of amino-acid-derived alcohols in the biosynthesis of lepidopteran pheromones has not been explicitly reported previously. However, the structures of isobutyl and (2*S*)-2-methylbutyl (7*E*)-7,9-decadienoate, components of the female produced sex pheromone of two *Darna* species (Lepidoptera: Limacodidae) (Sasaerila et al. 2000), strongly suggest valine and isoleucine to be precursors of the alcohol components. Some of the pheromone components of tussock moths, *Euproctis* spp. (Lepidoptera: Lymantriidae) are isobutyrate esters of various alcohols (Leonhardt et al. 1991; Wakamura et al. 1994; Yasuda et al. 1995), which again points to the involvement of valine, this time furnishing the acid part. By replacing malonyl CoA as the starting unit, branched-chain amino acids also are involved in the biosynthesis of methylalkanes after being transformed to activated branched carboxylic acids. Labelling experiments with crickets have revealed that valine serves as the starting material for biosynthesis of 2-methylalkanes with an even numbered carbon chain such as 2-methyltetracosane. The biosynthesis of uneven numbered chains as in 2-methylnonacosane starts from leucine (Blailock et al. 1976). Similar experiments have shown that 2-methylheptadecane, a sex pheromone of some arctiid moths, *Holomelina* spp. originates from leucine i.e., isovaleryl CoA (Charlton and Roelofs 1991), whereas the biosynthesis of 2-methyl-7*R*,8*S*-epoxyoctadecane, the female sex pheromone of the gypsy moth, *Lymantria dispar*, involves valine i.e., isobutyryl CoA (Jurenka et al. 2003).

**Fig. 5:**
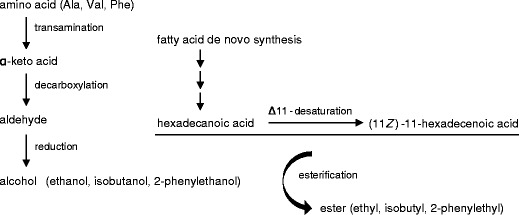
Proposed biosynthetic pathways for the esters in wings of male *Bicyclus martius sanaos*

To summarize, in the present study, we report on the identification of a series of new esters from the wings of male *B. martius sanaos* butterflies. We suggest that the biosynthesis of these esters (Fig. 5) occurs on the patches located on the forewings, in which hexadecanoic acid and the related (11*Z*)-11-hexadecenoic acid (or their activated forms) are esterified with ethanol, isobutanol, and 2-phenylethanol, respectively. The latter are derived from alanine, valine, and phenylalanine after a sequential process of transamination, decarboxylation, and reduction.

## Electronic supplementary material

Below is the link to the electronic supplementary material.

ESM 1 (PDF 9355 kb)
