# Supplementary material for: Identification and Biosynthesis of Novel Male Specific Esters in the Wings of the Tropical Butterfly, Bicyclus martius sanaos
Source: J Chem Ecol. 2014 Jun 4;40(6):549–59. doi: 10.1007/s10886-014-0452-y (PMC4090810; doi:10.1007/s10886-014-0452-y)
Supplement: Supplementary file 1 — (PDF 9355 kb) [file 10886_2014_452_MOESM1_ESM.pdf]

Supplemental information

Identification and biosynthesis of novel male specific esters in the wings of the tropical butterfly, *Bicyclus martius sanaos*

Hong-Lei Wang[1], Oskar Brattström[2], Paul Brakefield[2], Wittko Francke[3], Christer Löfstedt[1]

[1] Department of Biology, Lund University, Sweden

[2] Department of Zoology, Cambridge University, United Kingdom

[3] Institute of Organic Chemistry, University of Hamburg, Germany

Author for correspondence: hong-lei.wang@biol.lu.se

Department of Biology, Lund University, SE-223 62 Lund, Sweden

Legends for supplementary figures

Figure S1. Wings of male *Bicyclus martius*. **(a)** forewing on the ventral side. **(b)** hindwing on the ventral side. **(c)** forewing on the dorsal side. **(d)** hindwing on the dorsal side. **(e)** brushes on the dorsal side of forewing. **(f)** patch-like tissue on the ventral side of forewing. **(g)** brushes on the dorsal side of hindwing. Red circles indicate the sampling positions. The high-resolution photos were collected using a Leica DFC495 digital camera coupled to a Leica M125 stereomicroscope. Pictures were lightened and sharpened in Photoshop CS4.

Figure S2. Mass spectra of DMDS-adducts of monounsaturated esters. (a) DMDS-adduct of ethyl 11-hexadecenoate. **(b)** DMDS-adduct of isobutyl 11-hexadecenoate. **(c)** DMDS-adduct of 2-phenylethyl 11-hexadecenoate.

Figure S3. Mass spectra of 2-phenylethyl esters from wings of male *Bicyclus martius*. (a) 2-phenylethyl tetradecanoate (compound 6). **(b)** 2-phenylethyl octadecanoate (compound 10). **(c)** 2-phenylethyl octadecenoate (compound 9).

Figure S1

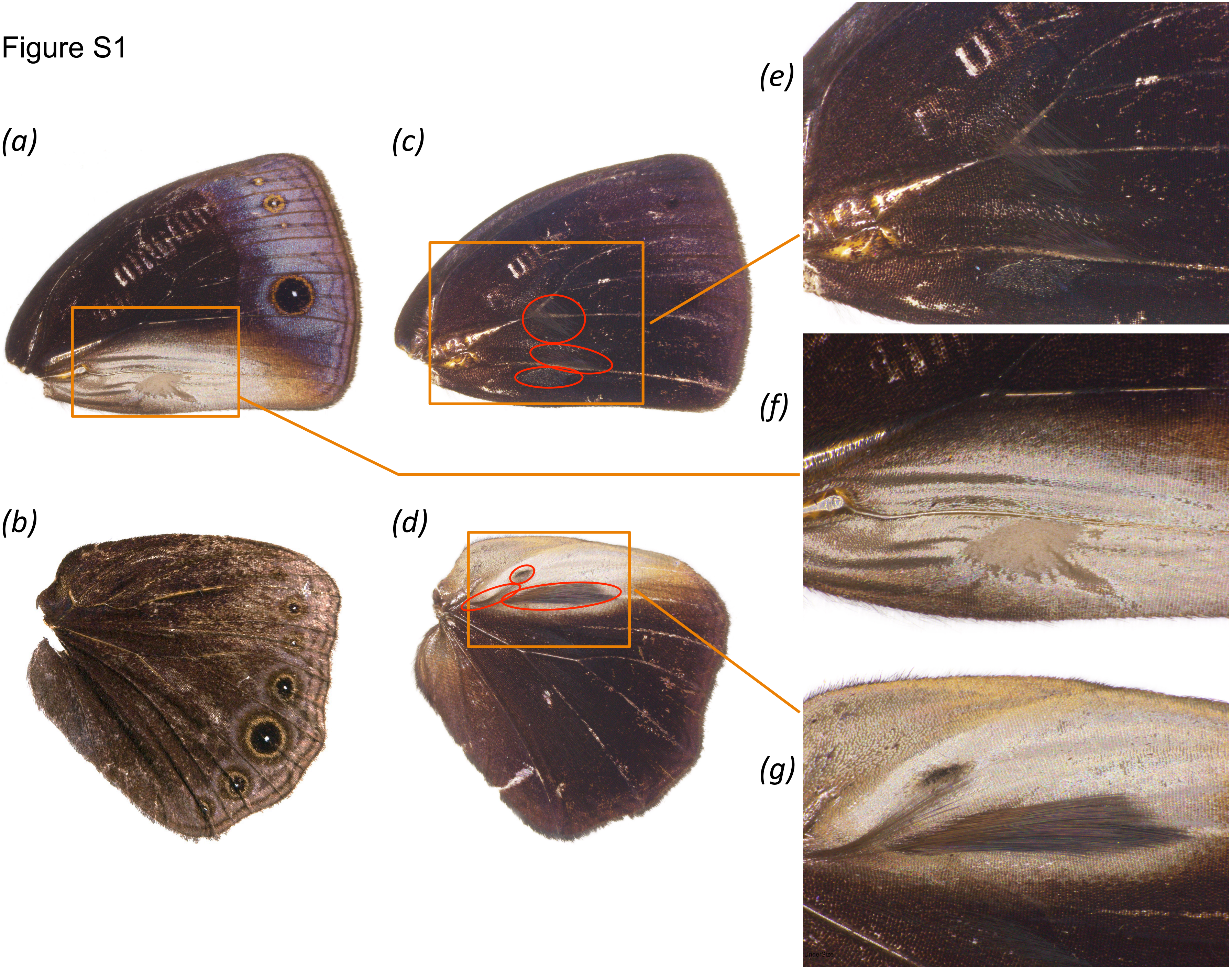

Figure S2

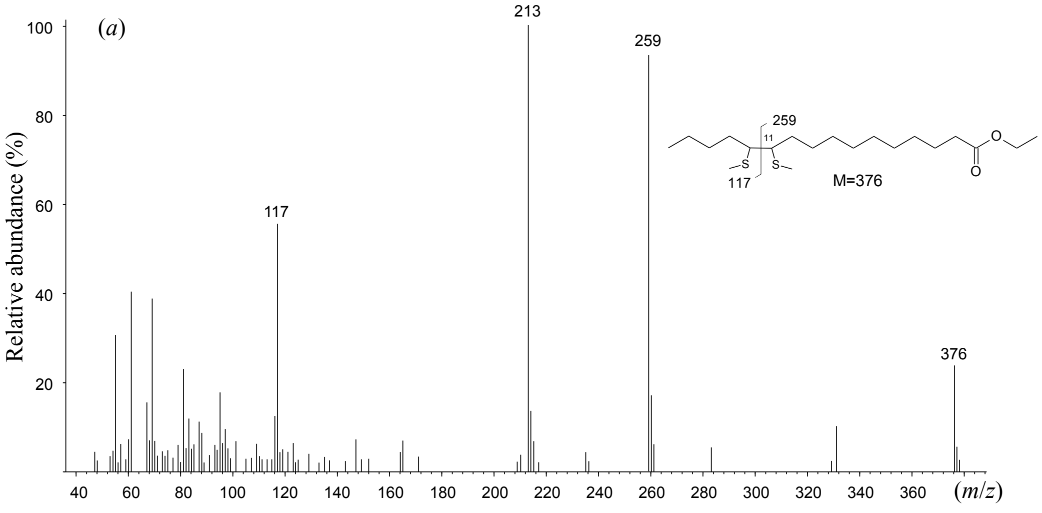

(a)
Relative abundance (%)
213
259
117
376
259
11
117
M=376
(m/z)

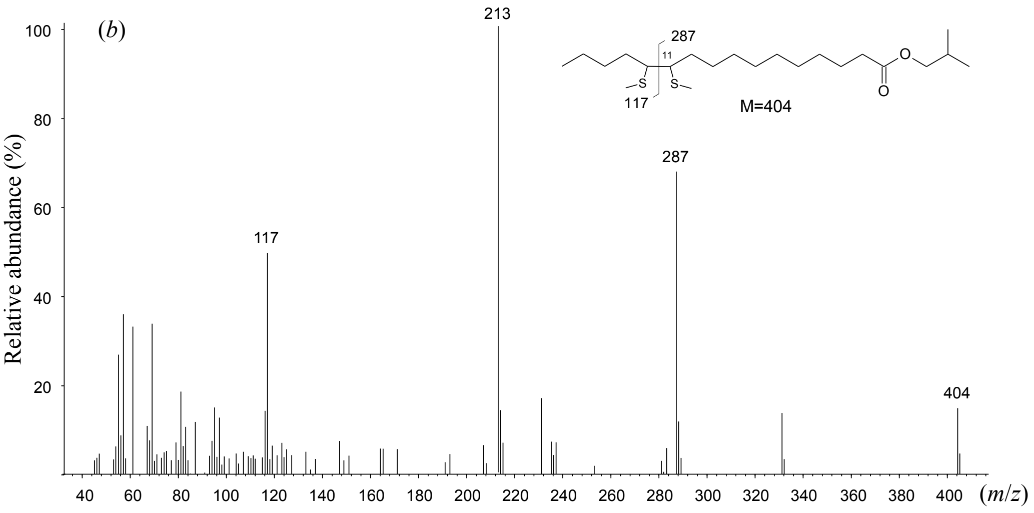

(b)
Relative abundance (%)
213
287
117
404
287
11
117
M=404
(m/z)

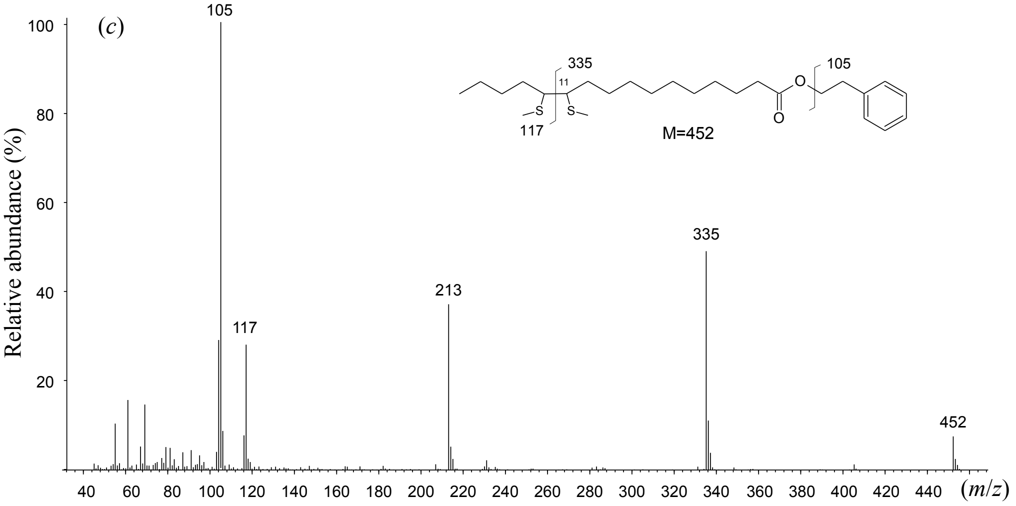

(c)
Relative abundance (%)
105
117
213
335
452
335
11
105
117
M=452
(m/z)

Figure S3

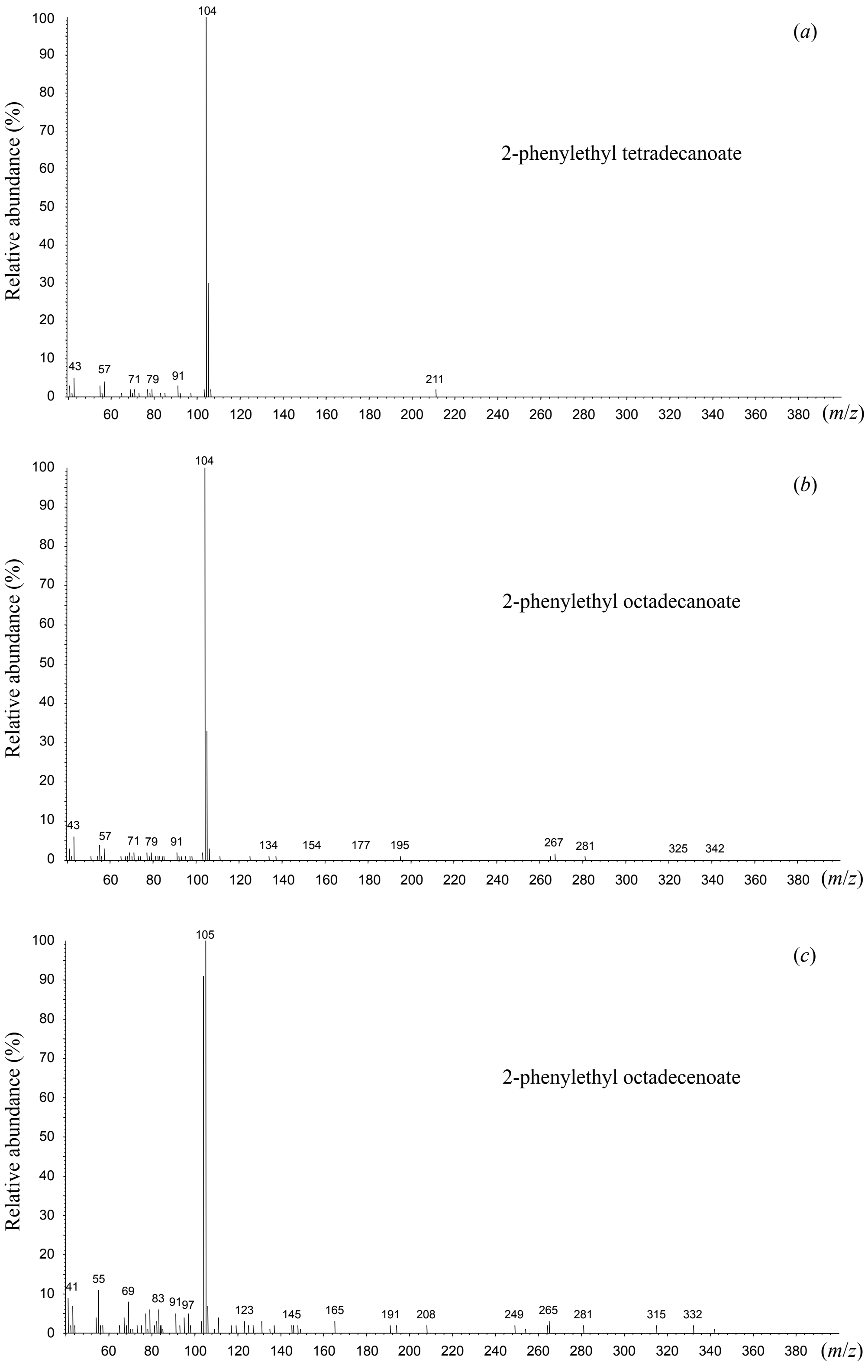
(a)
2-phenylethyl tetradecanoate
104
43
57
71
79
91
211
(b)
2-phenylethyl octadecanoate
104
43
57
71
79
91
134
154
177
195
267
281
325
342
(c)
2-phenylethyl octadecenoate
105
41
55
69
83
91
97
123
145
165
191
208
249
265
281
315
332
Relative abundance (%)
(m/z)
